# A CNN-based approach to riparian buffer strip quality monitoring in agricultural areas using satellite imagery

**DOI:** 10.1007/s10661-026-15760-w

**Published:** 2026-09-05

**Authors:** Samuel de la Sablonnière, Samuel Foucher, Yacine Bouroubi, Philippe Vigneault, Etienne Lord

**Affiliations:** 1 Saint-Jean-Sur-Richelieu Research and Development Centre, Agriculture and Agri-Food Canada, 430 Gouin Boulevard, Saint-Jean-Sur-Richelieu, QC J3B 3E6 Canada; 2 Department of Applied Geomatics, University of Sherbrooke, Sherbrooke, Canada

**Keywords:** Convolutional neural networks, Remote sensing, Riparian vegetation, Riparian strip quality index (RSQI)

## Abstract

Vegetated riparian buffers play a critical role in maintaining ecological health and water quality, yet efficient characterization and large-scale monitoring remain challenging due to the resource demands of traditional field campaigns. This study, conducted in an agricultural setting, introduces a straightforward, image-based methodology for riparian buffer characterization, exploiting advancements in deep convolutional neural networks (DCNN) and very high spatial resolution satellite imagery. Leveraging a large Riparian Strip Quality Index (RSQI) field dataset, the proposed approach adapts a Multi-View DCNN (MVDCNN) architecture, originally developed for 3D object recognition, to correlate satellite images of riparian strips with RSQI metrics. Of the seven spectral band combinations, multiple input views, and two training modes evaluated, the configuration using four views with RGB bands from a pretrained network achieved the best results. However, the alternative spectral band combinations produced similar levels of performance, suggesting that texture and shape information are key factors in the model’s effectiveness. Comparisons with a conventional workflow involving object-based land cover classification followed by RSQI calculation indicate that the trained MVDCNN achieves stronger correlations between imagery and RSQI scores (average RMSE = 7.35, R^2^ = 0.93 using RGB bands) compared to the object-based method (RMSE = 11.25, R^2^ = 0.87). To our knowledge, this is the first direct application of DCNNs to riparian buffer quality assessment. Requiring minimal preprocessing and no photo-interpretation expertise, the proposed approach leverages existing field data to facilitate more accessible, scalable, and adaptable riparian buffer monitoring, with potential for application in diverse environmental contexts.

**Supplementary Information:**

The online version contains supplementary material available at https://doi.org/10.1007/s10661-026-15760-w.

## Introduction

In agricultural environments, the intensive use of nutrients such as nitrogen and phosphorus in crop production compromises aquatic ecosystem structure and function (Carpenter et al., 1998; Collins et al., 2013), potentially having an adverse effect on biodiversity, which may depend upon the size of the water body (Rosset et al., 2014). Together with sedimentation, eutrophication through non-point source N and P enrichment may be especially important in lotic ecosystems within an agricultural setting. For example, 69% of the phosphorus load in the Rivière aux Brochets watershed in Quebec came from annual crops in 2003; the land upon which these crops were grown and harvested accounts for only 22% of the territory (Gangbazo et al., 2006).

Several beneficial management practices have been implemented to reduce these diffuse sources of pollution, including crop rotation, conservation of plant residues after harvesting, and the establishment of grassed swales. Riparian buffer strips, which are permanent natural vegetation strips along the edges of watercourses (Doyon, 2016; Lind et al., 2019), are a frequently proposed solution to mitigate these problems (Sosa et al., 2018a, b; Stutter et al., 2012). Although variable in terms of its degree of effectiveness (Dala-Corte et al., 2026; Trang et al., 2025), the riparian buffer is the last physical barrier (i.e., “last line of defense”) against downslope losses of materials from nearby soils to the receiving waterbodies. In addition to retaining nutrients and trapping sediment, the establishment of these buffers provides stabilization of the banks along the watercourse, together with its thermoregulation (shading provided by overhanging vegetation) and provision of biodiversity-based ecosystem services (Sosa et al., 2018a, b; Naiman & Décamps, 1997; Riis et al., 2020; Saint-Jacques & Richard, 1998; Schuft et al., 1999).

Worldwide, riparian buffer strips are subject to anthropogenic disturbances, including the adverse effects of urban sprawl, agricultural expansion, and the construction of levees and dams (Ghimire et al., 2025; Poff et al., 2011). Characterization of these ecotones (i.e., the transition zone between two adjacent ecosystems) and monitoring of their condition, therefore, is essential for providing an accurate assessment of their capacity to perform their ecological functions and to enable decision-makers to establish appropriate management and protection plans (Ashraf et al., 2010; Dufour et al., 2013; Trang et al., 2025).

Existing approaches for characterizing riparian strips are generally based upon the measurement of quality indices, such as the IBI (Index of Biological Integrity; Miller et al., (2006)), MQI (Morphological Quality Index; Rinaldi et al., (2017)) or QBR (*Qualitat del bosc de ribera*, in Catalan; Munné et al., (2003)), all of which have been developed specifically for this purpose. These indices include several indicators of ecosystem health (Pace et al., 2022), many of which are closely related to land-cover characteristics, such as the identification of plant communities, forest cover and channel slope. In Quebec (Canada), the Riparian Strip Quality Index (RSQI, which is described in Section "Riparian strip quality index (RSQI)") (Saint-Jacques & Richard, 1998) is used for this characterization. Overall, this index is a weighted sum of the areas that are occupied by different land-use components of the riparian zone, including forest, herbaceous plants, crops and built-up areas.

As demonstrated by Huylenbroeck et al., (2020), the use of remote sensing for characterizing riparian vegetation cover, which facilitates regional-scale applicability, has greatly increased in importance over the past decade. This is largely due to the reduced cost of acquiring Earth observation images, their increased availability, and improved spatial and spectral resolutions (Hemati et al., 2025). Various remote sensing products like Light Detection and Ranging (LiDAR) data, radar and multi-spectral images have been used for delineating riparian vegetation (Bertoldi et al., 2011), determining plant species composition (Macfarlane et al., 2017), and characterizing vegetation structure (Dufour et al., 2013; Hemati et al., 2025; Li et al., 2025; Michez et al., 2017; Petruželová et al., 2025), among others. For operational purposes, some studies have sought to establish more direct links between in situ characterization methods of riparian vegetation (i.e., measurement of indices) and remote sensing images.

In that sense and to permit assessment of riparian buffer strips on a regional scale, researchers in Andalusian Spain (Ivits et al., 2009) correlated the Green Vegetation Fraction (Gutman & Ignatov, 1998), which is calculated from Advanced Very High Resolution Radiometer (AVHRR) images, versus the ecological status of the riparian buffer (favourable vs unfavourable) in an agricultural environment. The results that were obtained are not very precise due to the very low spatial resolution of the images (1 km) and the use of only two classes. In the Cantabria region of Northern Spain, the Riparian Quality Index (RQI) (Del Tánago & De Jalón, 2011) was correlated with land-use maps that were produced from aerial photographs that had been interpreted by vegetation experts (Fernández et al., 2014). The best result had an *R*^2^ = 0.6 (r = 0.774), but implementation of the method required a high level of expertise. Pace et al., (2022) evaluated the capacity of remote sensing metrics to correlate with in-situ measurements of the QBR index, among other water quality indicators. From Sentinel-2 images (10 m resolution) and an available land-cover map, they found that the land use intensification index (LUI = 5 [%artificial] + 3 [%agricultural] + [%pasture]) at the reach scale achieved the best results, with a Pearson’s correlation r = −0.75 and a normalized RMSE of 0.34. In the Portneuf region of Quebec (Novoa et al., 2018), determination of RSQI was performed using object-based classification (OBC) of WorldView-2 images (50 cm resolution for the panchromatic band, 2-m resolution for the 8 multispectral bands). The RSQI that was produced by their classifier resulted in a mean of 56 versus 55 for the 80 in-situ measurements. Indeed, the authors demonstrated that satellite images with very high spatial resolution (< 1 m) contained the necessary information for the determination of an index such as the RSQI. Rivas-Fandino et al., (2022) employed a methodology similar to that of Novoa et al., (2018), also using WorldView-2 images. They correlated the computed RSQI with QBR that had been measured in the field, obtaining a Pearson’s correlation of 0.538 (*p* = 0.01). In the study by Saha et al., (2020), buffer strip widths of 25 m, 50 m and up to 500 m were evaluated for the computation of the RSQI from WorldView-2 images. These authors observed that buffers of 50 m width were optimal for correlation with the field-measured QBR index (Pearson’s r = 0.462, *p* < 0.001).

This type of study (as many others) relies upon field-based measurements of quality indices, which are mandatory for the purposes of validation and comparison. These measurements require extensive field data collection campaigns, expert aerial photo-interpretation or the production of detailed maps of the different plant species covering the territory. This process is costly in terms of time, human and financial resources, which limits the frequent recurrence of such undertakings. Still, in many countries around the world, it is conducted on an as-required basis, with the level of detail specified by local institutions. These large scale projects represent not only an important source of data for monitoring the quality of riparian buffer strips over time, but also potentially valuable reference datasets for training data-intensive models.

In recent years, Deep Convolutional Neural Networks (DCNN) have been applied to classification, scene recognition, segmentation and object-detection tasks on remote sensing images, given the excellent performance of this type of machine learning (Kussul et al., 2017; Ma et al., 2019; Romero et al., 2016). DCNNs do not require the intervention of experts to define the relevant attributes of objects or classes that are found in an image. They are hierarchically built, during the learning process, from the low-level features (e.g., textures, edges) to the highest levels (e.g., branches of a tree) (Nogueira et al., 2017). Yet, the complete training of a neural network requires a large amount of data (Scott et al., 2017), a prerequisite that is not always met in the field of Earth observation. This is why transfer-learning techniques (Pan & Yang, 2010) are generally used to mitigate this effect by using models pre-trained on large datasets as a training base. These include ImageNet (Deng et al., 2009) and PASCAL-VOC (Everingham et al., 2015). When it comes to exploring the possibilities of this type of model for determining the quality of riparian zones, the bottleneck is most frequently access to ground truth data containing a sufficient diversity and quantity of data. Given that field campaigns have occasionally been already conducted and made accessible locally, a modelling approach that is based on pre-trained DCNN, remote sensing images and the corresponding annotations (the quality index value) along watercourses can be explored.

Therefore, a Multi-View DCNN (MVDCNN) architecture that was originally developed for 3D object recognition was adapted to correlate images of a riparian strip that were extracted from satellite imagery with RSQI. The MVDCNN approach bypasses the segmentation and land cover classification steps required by traditional RSQI mapping workflows, enabling direct inference from satellite imagery. In addition to assessing the suitability of the approach, the performance of MVDCNN as a function of the spectral bands used was evaluated and compared with a more traditional method, namely object-based land cover classification and subsequent calculation of RSQI. Rather than focusing on achieving high benchmark scores or fine-tuning algorithms for optimal performance, this study emphasizes the presentation of a straightforward methodological approach that could be adapted to many types of environments and easily adopted by stakeholders. To our knowledge, this is the first direct application of DCNNs to the problem of riparian buffer characterization.

## Materials and methods

### Study area

**Fig. 1:**
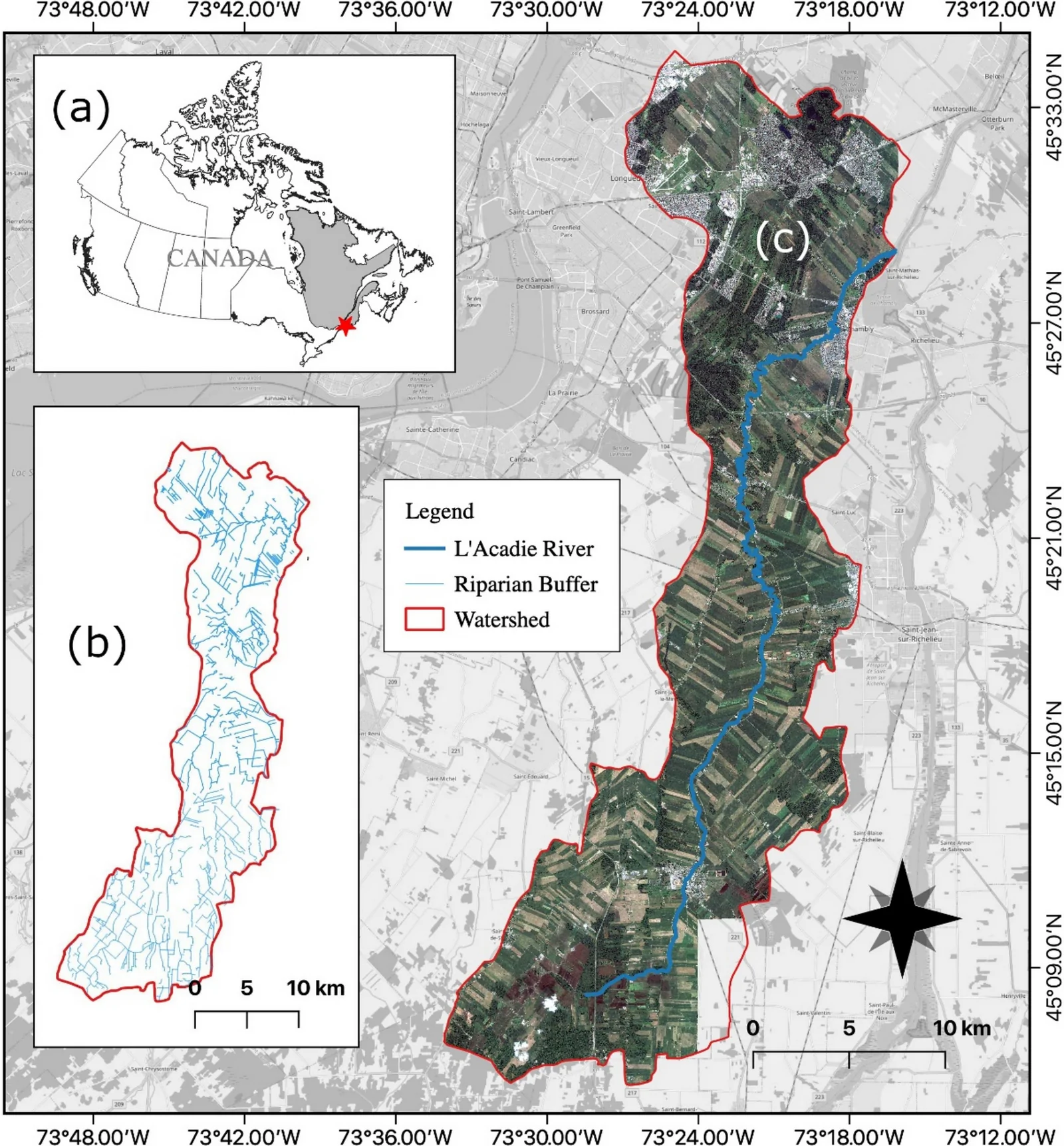
Study area location and data coverage. (**a**) Overview of Canada with Quebec province in gray and the study site marked by a red star; (**b**) L'Acadie River watershed (red) with riparian buffer strips shown as vector data (blue); (**c**) Pleiades satellite imagery mosaic (0.5 m resolution) with overlaid L'Acadie River vector data (blue)

The study area is the watershed of the L'Acadie River, which is a sub-basin of the Richelieu River, which is located in southern Quebec (Canada) (Fig. 1). With a surface area of 562.5 km^2^, it is the most important sub-basin of the Richelieu River. The L'Acadie River is 82 km long, with its source near the border with the United States of America (New York State), south of the village of Hemmingford, QC. The river ends its course as a tributary merging with the Richelieu River, north of the Chambly Basin.

The L'Acadie River watershed is largely dominated by agriculture, which accounts for 67% of its surface area (Covabar, 2015). The main crops that are grown in the watershed are rotations of maize (*Zea mays*) and soybean (*Glycine max*), which cover about 75% of the agricultural land that has been allocated to them. Wooded and urban sectors are few in number and account for 17% and 10% of the territory, respectively. The terrain is almost flat, except in the extreme northern portion of the watershed, where Saint-Bruno Mountain (one of the nine recognized Monteregian Hills) rises to 218 m.

As for riparian zones specifically, their composition reflects the agricultural dominance of the watershed, consisting primarily of herbaceous vegetation, trees and shrubs, bare soil, and cropland. The predominantly flat terrain results in very few steep-sloped banks, and rocky areas are almost entirely absent. Urban riparian zones are limited to the northern sector with few instances, and there is no active deforestation in the watershed. This landscape context is characteristic of southern Quebec agricultural lowlands and represents the environmental conditions on which model training and validation were based.

### Riparian strip quality index (RSQI)

The RSQI is a weighted sum of the areas that are occupied by the land-use components of the riparian buffer strips, where the greatest weights are assigned to the surface types contributing most to the health of the watercourse. It assesses “the ecological condition of the riparian habitat and its impact on the integrity of the aquatic environment” (Saint-Jacques & Richard, 1998). It is defined according to the Eq. (1):

1 $$RSQI=\frac{\sum\left(\mathrm{\%}_{i}\times P_{i}\right)}{10}
$$

where *%*_*i*_ is the percentage ground cover of the *i*^*th*^ component of the riparian buffer, and *P*_*i*_ is the weighting factor for that component. A width of 10 m of riparian buffer is considered in the calculation of the areas over a length that depends upon the bank sections being determined. More specifically, if a significant change in land use is observed, e.g., a change from urban to forest, a new section is delineated. The weighting factors are: forest (10.0); shrub (8.7); herbaceous (5.8); forest cut (4.3); rocky soil (3.8); pasture/agricultural grassland (3.0); crop (1.9); infrastructure (1.9); and bare soil (1.7). RSQI index values, therefore, range from 17 to 100, with the highest value representing the best condition in terms of the capacity of the riparian buffer to fulfil its ecological functions. The RSQI can be classified as follows: very poor (17–21); poor (21–40); average (40–60); good (60–80); and excellent (90–100) (Covabar, 2015).

**Fig. 2:**
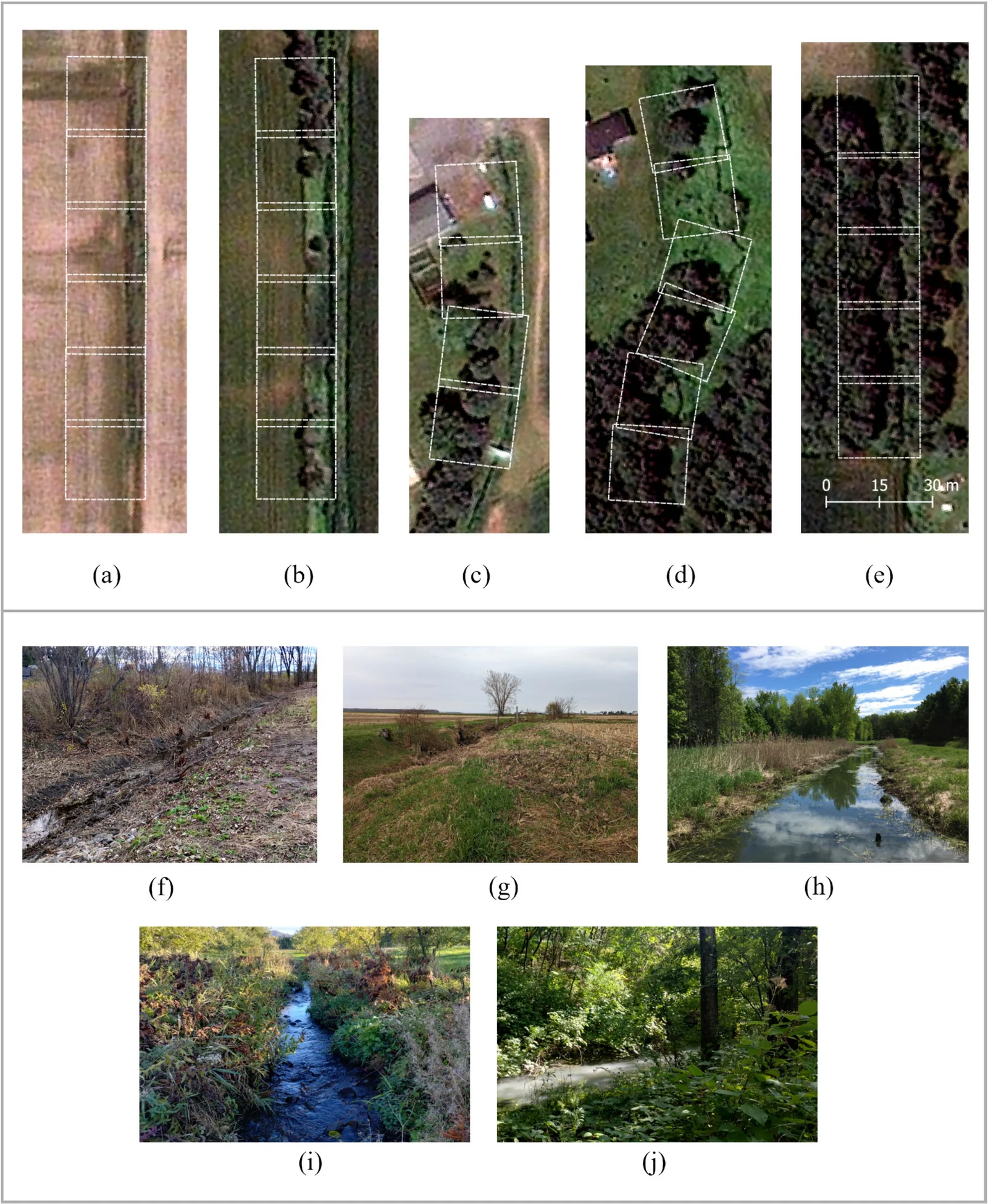
Examples of riparian strips for different values and classes of RSQI. (**a–e**) Pléiades image (0.5 m pixel resolution) showing RSQI values of (**a**) 19 (very poor), (**b**) 45 (poor), (**c**) 65 (average), (**d**) 79 (good), and (**e**) 100 (excellent). The footprint of each image patch extracted from the Pléiades mosaic is represented by white squares, centered 10 m from the watercourse, with dimensions of 23 m × 46 pixels per side. (**f**–**j**) Ground photographs from different locations illustrating visual examples of very poor, poor, average, good, and excellent RSQI classes, respectively

In agricultural areas, a riparian buffer with a very low RSQI is characterized by the virtual absence of natural vegetation along the watercourse (Fig. 2a, f), while a certain width of vegetated buffer would be present for a “poor” class (Fig. 2b, g). In the “average” class, trees and natural vegetation are present to a greater extent along the riparian buffer, but these are often sparse and accompanied by built-up areas (Fig. 2c, h). High values represent riparian buffers where natural forb (non-graminaceous herb) and forest cover dominates (Fig. 2d, e, i, j). This assessment protocol has been widely adopted by the entities that are responsible for assessing the state of riparian ecosystems in the Province of Quebec (Canada). Although it is measurable through the use of photo-interpretation, the RSQI is measured directly, in practice, in the field by experts.

### Field data

The field data collected between 2012 and 2015 during the characterization of the L’Acadie River’s tributaries were provided by the COVABAR watershed protection organization. These data represent 697 km of watercourses that were surveyed by multiple teams (see riparian strips in vector format in Fig. 1). The reference vector data, which designate the watercourses, are based on public mapping information provided by the Governments of Quebec and Canada. The riparian buffer strips are divided into homogeneous sections of varying length, based upon compositional changes that were observed in the field. The parameters needed to calculate RSQI were estimated by the evaluators for each of the riparian sections, and the RSQI value was then computed in Excel spreadsheets. The dataset provided was in a shapefile format, where each vector segment contains the RSQI value measured for each bank of the watercourse.

### Satellite images

The Pléiades constellation comprises two optical Earth observation satellites (PHR 1 A and PHR 1B) operated by CNES and Airbus Defence and Space. Three PHR 1B images were acquired for this study: two from 6 July 2013 and one from 8 July 2015, providing spatial coverage of the area. Only a limited portion of the southeastern study region (see Fig. 1) remains uncovered by these datasets. The spectral bands used included Blue (B, 430–550 nm), Green (G, 490–610 nm), Red (R, 600–720 nm) and Near-infrared (NIR, 750–950 nm) with a spatial resolution of 2 m, together with a panchromatic band (480–830 nm) with a resolution of 0.5 m. The images were resolution enhanced by the PCI Geomatics Catalyst (version 2222.0.4) PANSHARP algorithm and subsequently orthorectified using a 1-m resolution digital elevation model (DEM), which was derived from LiDAR data. The radiometric resolution was reduced from 12-bit to 8-bit to facilitate training of the neural networks. Following pre-processing, an RGB-NIR mosaic of the study region at 0.5-m resolution was produced (see Fig. 1).

### Automated dataset creation

**Fig. 3:**
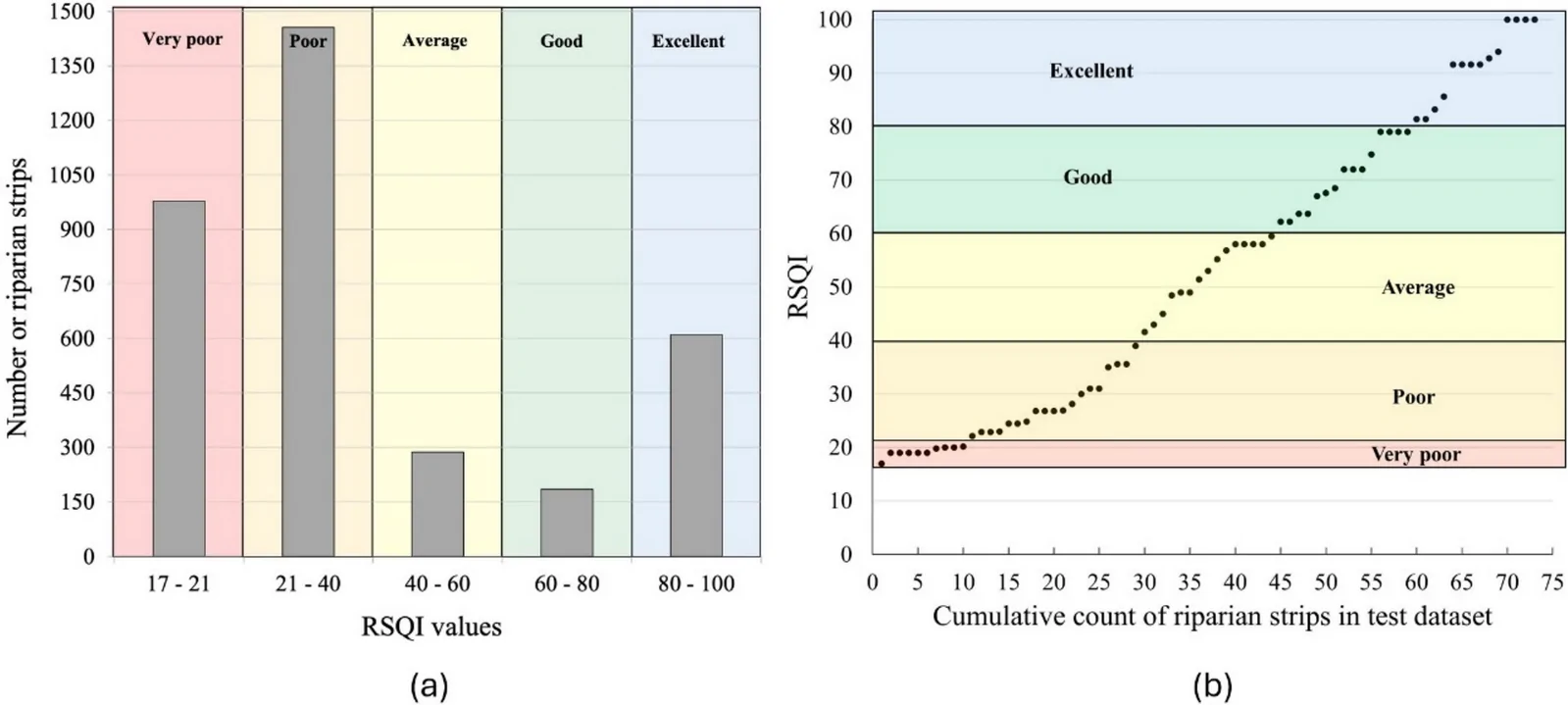
Distribution of RSQI values in the entire dataset (*N* = 3,489 riparian strips), in (**a**) according to quality class and in (**b**) distribution of the riparian strips composing the test dataset (*N* = 75 riparian strips)

Image patches were extracted with a size of 46 pixels on a side (23 m) with a centre distance of 10 m from the nearest water stream (see Fig. 2). Annotation was performed automatically by assigning each image patch the RSQI value obtained from the shapefile at the corresponding location. The image patch size and center offset were determined empirically to ensure sufficient contextual information for the DCNN while avoiding the inclusion of objects too distant from the riparian strip. With a theoretical riparian buffer width of 10 m for RSQI calculation, the 23 m patch centered 10 m from the stream ensures complete buffer coverage (extending slightly into the water) while capturing adjacent landscape context. In total, 46,209 image patches were sampled and grouped into 3,489 riparian strips. Figure 3a shows the distribution of RSQI indices evaluated in the field dataset.

To provide a balanced and curated test dataset for model evaluation, 75 riparian strips were randomly selected from the mosaic, ensuring coverage of the full range of RSQI values, and removed from the training and validation processes (see Fig. 3b). Particular attention was paid to the reliability of RSQI values relative to what was observable in the imagery. Furthermore, to properly isolate the test set from the training set, riparian buffers from both river banks were retained in the test set when they exhibited similar land-cover composition. This precaution accounts for the fact that opposite banks of a given stream reach often show visual similarities. Similarity between banks was assessed both visually and quantitatively by comparing RSQI values. The relatively small size of this test set (2% of the riparian strips) reflects the need to maintain balance across RSQI values while retaining sufficient samples in each RSQI class for training (see Fig. 3). As detailed in *Training protocol* section, an additional 20% of riparian strips were used for validation, resulting in an even smaller number of examples remaining in the “average” and “good” RSQI classes for training.

### MVDCNN architecture

In most cases, a DCNN learns from a label that is associated with an image, with either a category (e.g., maize or wheat) or a numerical value. Characterization indices, in contrast, are measured over strips of land varying in length from a few tens of metres to more than one km. Thus, a single image patch is not sufficient to represent the riparian strip and its index (the label).

In the field of three-dimensional (3D) shape recognition, a multi-view DCNN-based architecture (MVDCNN) has been proposed (Su et al., 2015). Its main principle is to synthesize the information that is included in multiple images into a single compact descriptor. The architecture consists essentially of the VGG-M DCNN (Chatfield et al., 2014), to which the researchers have added a view-pooling layer immediately following the last convolutional layer. During training, different images of the same object, called views, are passed sequentially through an encoding network, and the embeddings generated for each image are accumulated up to the view-pooling layer. The principle of this layer is very similar to a max pooling layer (Krizhevsky et al., 2012) that is frequently used in DCNN, except that the operation is performed along the view axis. The main advantage of this architecture is its flexibility: since the number of views at the network input does not affect the network architecture, it can be adjusted according to the user's needs. It also remains computationally efficient, as the same encoding backbone is applied to all input views.

**Fig. 4:**
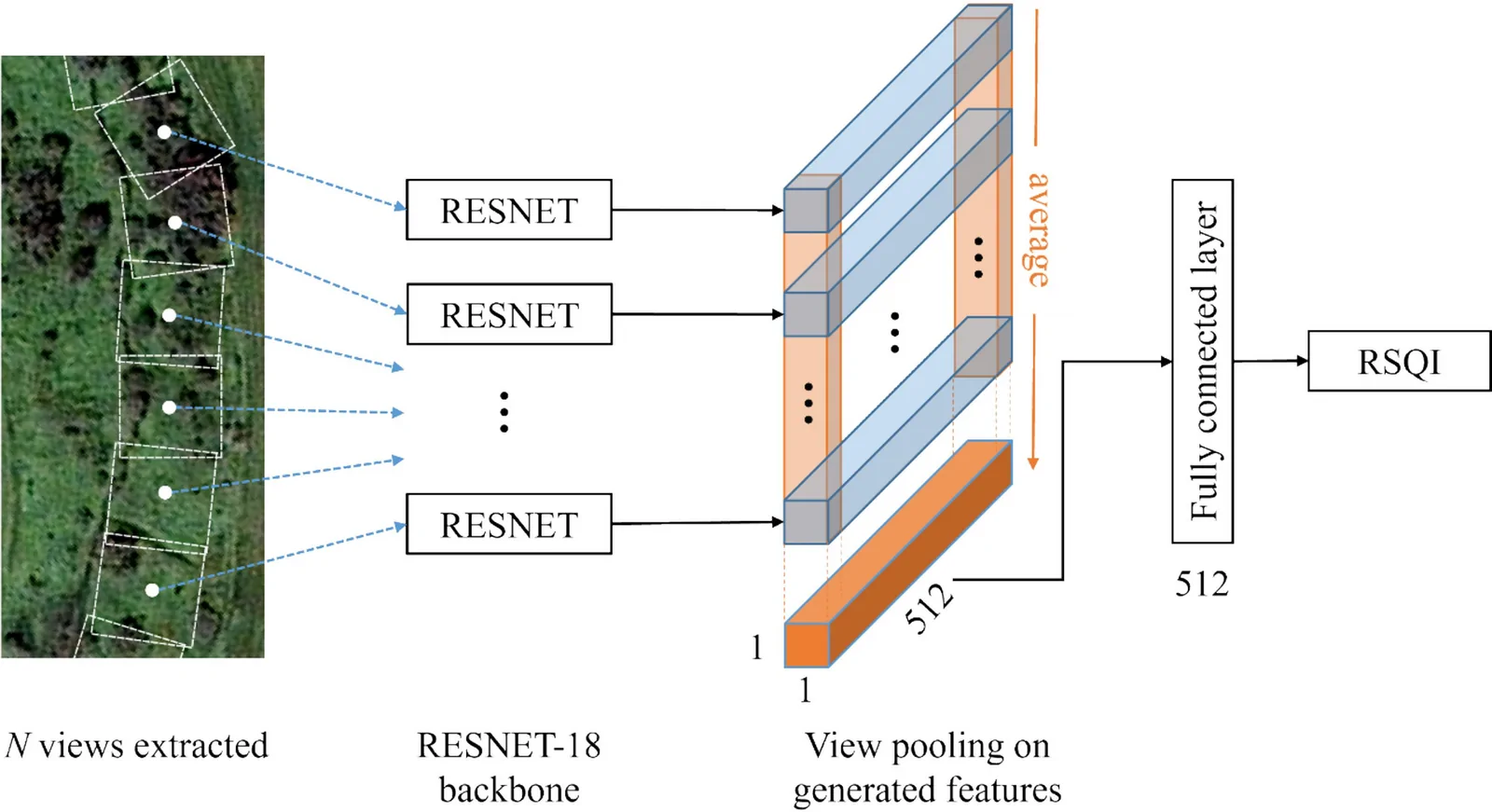
MVDCNN architecture used for the determination of RSQI, as inspired by Su et al. (2015). A set of (N) RGB views extracted from the Pleiades mosaic are used as input to the MVDCNN. Averaging is performed in the view-pooling layer. The output is the predicted RSQI value

From an ecological perspective, we hypothesize that this architecture should allow the model to integrate spatially distributed information along the riparian strip. Each image patch extracted from the Pléiades mosaic—referred to hereafter as a "view" following MVDCNN terminology—captures local conditions at a specific position, while the view-pooling layer synthesizes these local assessments into a global representation of riparian quality. This design choice is in line with the fact that RSQI values emerges from the aggregate conditions along the strip, rather than from any single location. In this paper, an architecture that was based on MVDCNN was repurposed and is presented in Fig. 4. In our implementation, the VGG-M network was replaced by the RESNET-18 architecture (He et al., 2016), as the latter has fewer parameters while providing superior training efficiency and classification performance compared to VGG-M (He et al., 2016). Because RSQI is a weighted average, the view-pooling layer in our model uses the average operator rather than the maximum, which was used in the original MVDCNN design. During training, riparian strip views are input in arbitrary order into the RESNET-18 backbone, and their embeddings are averaged in the pooling layer (see Fig. 4). The resulting features are then passed to a fully connected layer with 512 nodes, from which the RSQI prediction is produced as the final output.

## Network training and evaluation

This section describes the systematic evaluation of input configurations and spectral information for training the MVDCNN network to predict riparian strip quality. The procedure includes determining the number of views used as network input, evaluating different combinations of spectral bands, and applying two training strategies. The data preparation, training protocol, and validation process are presented to provide a clear framework for assessing model performance.

### Determining the number of views

In order to determine the optimal number of input views for training, several core components of network configuration, including spectral band selection and training strategies, were incorporated into preliminary evaluations. These aspects are outlined in the following sections, but are introduced here as they are integral to the selection process.

Specific numbers of views of the riparian buffer strips, namely 1, 2, 4, 8, 12 and 16 were evaluated as simultaneous inputs to the network for RSQI prediction. The same training set was used throughout; however, each riparian strip is represented by a specific number of views (from 1 to 16), and these strips may include several dozen patches in total. This replication is necessary in order to use a fixed batch size during the training phase. For comparison, using a single input view is equivalent to training a standard RESNET-18, as the view-pooling layer then averages over only one value. In these experiments, the MVDCNN was trained starting from an ImageNet pre-trained RESNET-18 (see Training protocol section for details), with training images composed exclusively of RGB visible bands. Once the optimal number of input views was established, this configuration was used for all subsequent training sessions.

### Training protocol

**Fig. 5:**
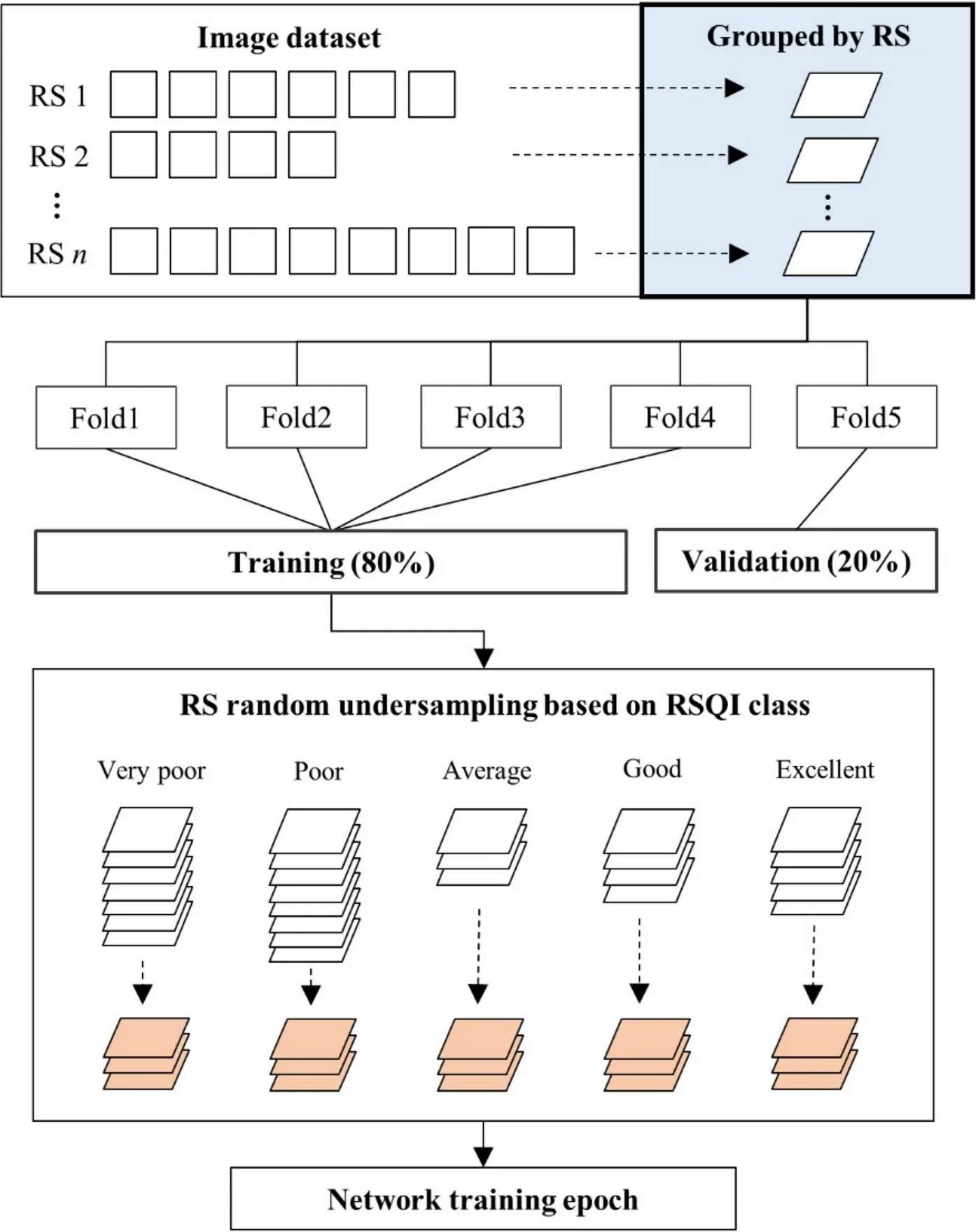
Flowchart used in preparing the image dataset for network cross-validation training. First, the views are grouped by riparian strip (RS). Second, the set of riparian strips is split into 5 folds and the preliminary training set is formed. Third, under sampling of RS is performed according to each RSQI class, which constitutes the training dataset upon which the network learns

All training was performed with a fivefold cross-validation protocol (see Fig. 5). The dataset was randomly split into five folds, each containing between 599 and 634 Riparian Strips (RS). For each of the five training cycles, four folds (80%) were used for training and one fold for validation (20%). Random under-sampling was applied to address class imbalance in the RSQI distribution (see Figs. 3a and 5). To make optimal use of the dataset, random sampling of views from each RS (i.e., the 1–16 patches per riparian strip, as described above) was performed at each training epoch (i.e., a complete forward and backward pass through the data). For all network training, data augmentation techniques such as image rotation (0–45°) and flipping were used to artificially increase dataset size and reduce the risk of overfitting. Figure 5 illustrates the preparation of the dataset for network training.

To assess the effect of the spectral bands used, several combinations of bands were evaluated: RGB, RGN, RGBN, as well as single repeated spectral bands (RRR, GGG, BBB, NNN). In these latter cases, each spectral band was replicated across all three input channels, allowing the use of pre-trained models designed for three-band input while evaluating the discriminative power of each spectral band independently. Two training strategies were employed: fine-tuning the MVDCNN with a pre-trained RESNET-18 on ImageNet (except for the RGBN composite), and training from scratch. While fine tuning, only the top two convolutional layers and the fully connected layer were updated, as this approach yielded the best results in previous studies on the UCMerced (Yang & Newsam, 2010) and RS19 (Xia et al., 2010) satellite image datasets (Nogueira et al., 2017). The cost function used for gradient back-propagation was the mean-square-error (MSE) and the optimizer was Adam (Kingma & Ba, 2015). The learning rate was set to 0.001 for training from scratch and 0.0001 for fine-tuning; batch size was 128, and the number of training epochs was set to 60 for both strategies.

### Validation

The validation dataset (1 of the 5 folds) was used to verify the accuracy of the selected hyperparameters and to evaluate the performance of the MVDCNN during training. To reduce randomness in the validation phase (performed at each training epoch), all views from each riparian strip (rather than a fixed number used in training) were used as network input with a batch size of 1. This allowed the MVDCNN to make predictions on a variable number of input views, depending on the length of the riparian strip. The network that achieved the best performance at a given training epoch was selected for the test phase. No image augmentation techniques were applied during this final phase.

### Network testing

The test dataset, consisting of 75 randomly distributed riparian strips (RS), was used to evaluate network performance. The employed performance metrics were the root-mean-square error (RMSE) and the coefficient of determination (R^2^). RMSE measures the average error between model predictions and observed values and is defined as follows:

2 $$RMSE=\sqrt{\frac{1}{N}{\sum}_{i=1}^{N}{\left({y}_{i}-\widehat{{y}_{i}}\right)}^{2}}$$

where N is the number of observations, ${y}_{i}$ is the observed (ground-truth) value, and ${\widehat{y}}_{i}$ is the predicted value for the i-th observation. R^2^ represents the proportion of variance in the dependent variable explained by the independent variables in the regression model. It is defined as:

3 $${R}^{2}=1-\frac{{\sum}_{i=1}^{N}{\left({y}_{i}-\widehat{{y}_{i}}\right)}^{2}}{{\sum}_{i=1}^{N}{\left({y}_{i}-\overline{y}\right)}^{2}}$$

where N is the number of observations, ${y}_{i}$ is the observed value, ${\widehat{y}}_{i}$ is the predicted value, and $\overline{y}$ is the mean of observed values. For each metric, the mean and standard deviation across the five cross-validation runs are reported. Results for the validation fold are also provided, along with results for a balanced variant of this fold (balanced by RSQI class) to enable a more accurate assessment of model performance during validation.

To further evaluate the robustness of the trained model, two additional tests were performed: (1) prediction of RSQI for a randomly generated riparian strip assembled from views within the test set, and (2) prediction of RSQI as a function of the starting position of the extracted views relative to a simulated stream. The first test, which makes inferences using a random selection of distinct riparian buffer strip views from the test dataset, assesses the network’s ability to account for inter-image variability. The second test evaluates sensitivity to intra-image variability by modulating the proportion of forest and agricultural land present within each view composing a riparian buffer.

All experiments were performed using the Pytorch 1.5.0 library using python v3.7. Training and testing were performed on a computer with a 3.5 GHz Intel Core i7-5930 k processor, 64 GB of RAM and a NVIDIA GeForce GTX 1080 graphics card.

### Object-based classification

To compare the performance of the developed model with a more traditional approach, we computed the RSQI for the riparian strips in our dataset using an object-based land cover classification method similar to that described by Novoa et al., (2018). This was accomplished using the Object Analyst module from PCI Geomatics Catalyst (version 2222.0.4). For the segmentation step, a scale parameter of 30 was used, while the shape and compactness parameters were both set at 0.2. These settings enabled the effective delineation of elongated objects, such as the herbaceous edges commonly found along riparian strips. In total, 186,936 objects were segmented within a 50 m buffer surrounding the watercourses in the field dataset.

Following the segmentation step, attribute calculation was performed to extract statistical information for each segmented object. Twenty-two attributes were retained, including the minimum, maximum, and standard deviation of the four spectral bands, as well as contrast and entropy layers generated for this purpose. Geometric attributes such as elongation, circularity, compactness, and convexity were also considered. For the classification step, objects were manually selected as training and validation samples for each of the nine land cover classes included in the RSQI (see Riparian Strip Quality Index section above). Since the “forest cut” and “rocky soil” classes were not present at the study site, they were excluded. An additional “other” class was created to represent water and inland shoreline areas. A total of 100 validation samples were collected using a stratified sampling approach with proportional allocation, with sample sizes ranging from 6 to 20 per class. This approach balanced the need for statistical reliability across all classes while accounting for their relative landscape prevalence, ensuring that even rare classes (shrub and other, n = 6 each) had sufficient representation for accuracy assessment. Finally, a support vector machine (SVM) classifier was used to classify the segmented objects. A confusion matrix was generated, and a kappa coefficient (Cohen’s κ) was calculated to evaluate the agreement between ground truth and predicted land cover classes on the validation objects (Cohen’s κ: 1 = perfect agreement, 0 = agreement by chance, −1 = complete disagreement).

For RSQI calculation, a 10-m wide riparian buffer was considered. For each riparian buffer strip in the dataset, the number of pixels corresponding to each land cover class within this buffer was determined, and the RSQI value was calculated using Eq. (1). Pixels classified as “other” were not included in the calculation. These RSQI values were then compared to those predicted by the proposed model.

## Results

### Determining the optimal number of input views

**Fig. 6:**
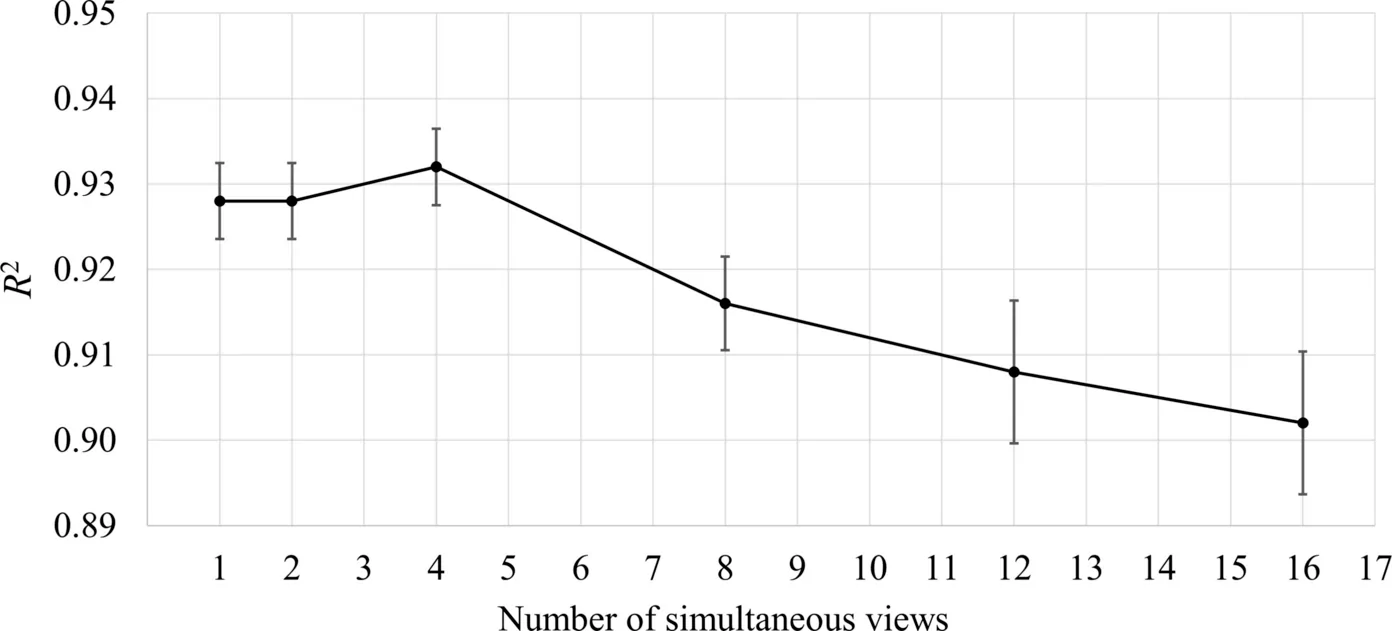
*R*^2^ results of RSQI prediction on the test set as a function of the number of input views (1 to 16). Best results were obtained with a set of 4 views per riparian strip. Training was performed with the RGB spectral bands with fivefold cross-validation. Error bars represent standard deviations from five independent trainings of the MVDCNN incorporating a RESNET-18 pretrained on ImageNet

**Fig. 7:**
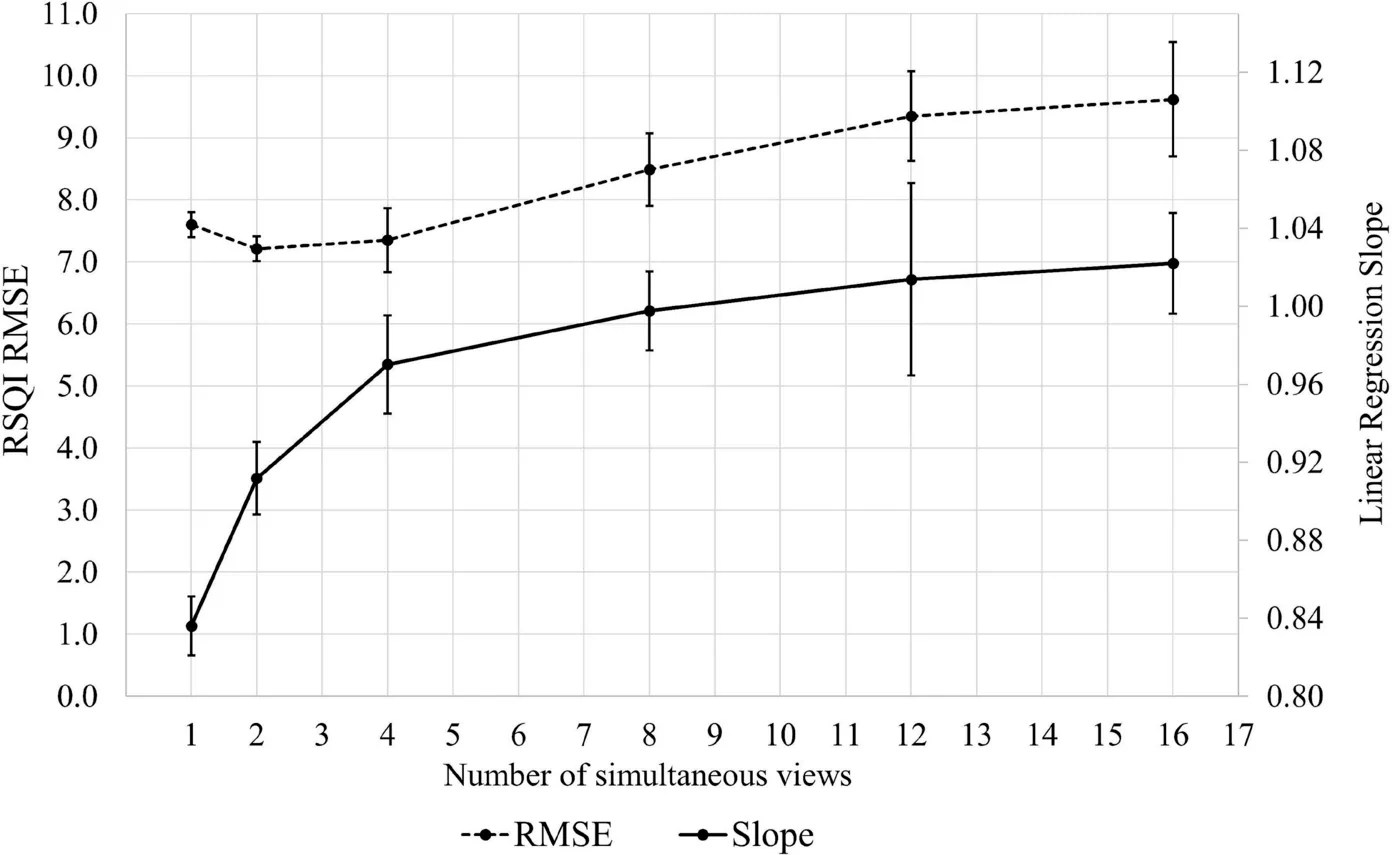
RMSE (Root Mean Square Error, dotted line, left y-axis) and regression slope (b, solid line, right y-axis) obtained on the test set as a function of the number of simultaneous input views. The most balanced results (low RMSE and regression slope close to 1.0) were obtained with 4 input views. Training was performed using the RGB spectral bands. Error bars represent standard deviations of five independent trainings of the MVDCNN incorporating a RESNET-18 pretrained on ImageNet

During training, different numbers of views were evaluated to determine the optimal value. In other words, the number of views representing each riparian strip in the dataset was varied before being provided as input to the network. The results are shown in Figs. 6 and 7.

When comparing RMSE values of predicted RSQIs as a function of the number of input views, results ranged from 7.21 ± 0.20 for two input views to 9.62 ± 0.92 for sixteen input views, with corresponding R^2^ values of 0.928 and 0.902, respectively. For a single input view, the RMSE and R^2^ were 7.6 ± 0.20 and 0.928, respectively. However, the regression slope for a single view (b = 0.836) deviated the most from the ideal 1:1 slope (b = 1.0) among all configurations, compared to b = 0.970 using four views. Low RSQI values (17–21) are overestimated, while high RSQI values (85–100) are underestimated. The most balanced trade-off between RMSE and regression slope was achieved with four input views, with RMSE = 7.35 ± 0.52 and b = 0.970. Furthermore, this configuration yielded both the highest average R^2^ (0.932) and the lowest RMSE (6.95) across the five cross-validation runs. Beyond four views, adding more views consistently increased RMSE and decreased model stability (as shown by higher standard deviations). Consequently, the four-view configuration was retained for all subsequent experiments.

### Effect of spectral bands choice

Seven spectral band combinations were tested using two training strategies. RMSE and *R*^2^ obtained on the validation fold ("as is" and balanced) and the test dataset are shown in Tables 1 and 2, respectively.

**Table 1:** Mean RMSE (with standard deviations) of RSQI predictions across five cross-validation runs on the validation, balanced validation, and test datasets, shown for both training strategies and all seven spectral band combinations

	From scratch			Fine-tuned^2^		
Spectral bands	Validation	Balanced validation	Test	Validation	Balanced validation	Test
RRR	11.49 ± 0.68	12.71 ± 0.61	8.56 ± 0.28	11.95 ± 0.52	12.64 ± 1.11	8.02 ± 0.36
GGG	11.58 ± 0.52	13.02 ± 0.80	**8.18 ± 0.31**	11.72 ± 0.61	13.03 ± 0.64	8.27 ± 0.30
BBB	11.22 ± 0.37	12.69 ± 0.61	8.35 ± 0.26	11.67 ± 0.44	12.82 ± 1.06	8.04 ± 0.37
NNN	11.79 ± 0.63	12.93 ± 1.13	8.37 ± 0.22	11.73 ± 0.45	12.70 ± 0.93	9.10 ± 0.20
RGB	11.68 ± 0.77	12.66 ± 0.29	8.83 ± 0.29	11.69 ± 0.75	12.56 ± 1.23	**7.35 ± 0.52**
RGN	11.63 ± 0.85	12.65 ± 0.61	8.86 ± 0.52	11.39 ± 0.59	12.57 ± 0.75	7.76 ± 0.24
RGBN	11.28 ± 0.82	12.68 ± 0.31	8.94 ± 0.58			
OBC classifier	13.65 ± 0.53	15.11 ± 0.64	11.25^1^			

^1^In the case of the object-based classification (OBC) on the test set, only the best model was used for the RSQI determination. ^2^The fine-tuned network was only used with combinations of 3 spectral-bands

Results from RSQI calculations using the object-based classification (OBC) method are also presented. The best results for each training method are highlighted in bold

**Table 2:** Average R^2^ values (with standard deviations) of RSQI predictions across five cross-validation runs for the validation, balanced validation, and test datasets, shown for both training strategies and all seven spectral band combinations

	From scratch			Fine-tuned^2^		
Spectral bands	Validation	Balanced validation	Test	Validation	Balanced validation	Test
RRR	0.846 ± 0.018	0.798 ± 0.021	0.898 ± 0.008	0.840 ± 0.012	0.795 ± 0.030	0.916 ± 0.009
GGG	0.836 ± 0.018	0.790 ± 0.023	**0.908 ± 0.008**	0.838 ± 0.013	0.791 ± 0.012	0.908 ± 0.005
BBB	0.848 ± 0.015	0.801 ± 0.016	0.902 ± 0.008	0.842 ± 0.016	0.797 ± 0.027	0.914 ± 0.009
NNN	0.836 ± 0.021	0.795 ± 0.028	0.906 ± 0.006	0.838 ± 0.016	0.799 ± 0.025	0.890 ± 0.007
RGB	0.840 ± 0.019	0.804 ± 0.011	0.900 ± 0.007	0.842 ± 0.022	0.804 ± 0.033	**0.932 ± 0.005**
RGN	0.842 ± 0.022	0.803 ± 0.017	0.892 ± 0.013	0.844 ± 0.015	0.805 ± 0.018	0.924 ± 0.006
RGBN	0.848 ± 0.020	0.803 ± 0.015	0.892 ± 0.008			
OBC classifier	0.795 ± 0.008	0.732 ± 0.027	0.871^1^			

^1^In the case of the object-based classification (OBC) on the test set, only the best model was used for the RSQI determination. ^2^The fine-tuned network was only used with combinations of 3 spectral-bands

Results from RSQI calculations using object-based classification (OBC) are also presented. The best results are shown in bold

As shown in Table 1, all configurations trained from scratch yielded very similar results on the test dataset, with average RMSEs ranging from 8.18 ± 0.31 for the GGG bands to 8.94 ± 0.58 for the RGBN bands. Results using a single repeated spectral band (RRR, GGG, BBB, or NNN) were superior to those achieved with compound spectral bands (RGB, RGN, RGBN). Overall, these findings suggest that the network does not rely on diverse spectral information to converge. A similar trend is observed in Table 2, where the best R^2^ was obtained with the GGG bands (0.908), while the lowest R^2^ corresponded to the combined use of the four RGBN bands (0.892). Interestingly, the RGB configuration yielded a higher R^2^ (0.90) than the RGN bands (0.892), which was unexpected given that the B-band is generally considered less informative for vegetation characteristics. In summary, the network achieved consistently good results regardless of the spectral bands used when trained from scratch.

When fine-tuning a pre-trained RESNET-18, RMSEs on the test dataset ranged from 7.35 ± 0.52 and 7.76 ± 0.24 for the RGB and RGN bands, respectively, to 9.10 ± 0.20 for the NNN bands. Results using the RGB and RGN spectral bands outperformed all repeated single-band configurations. The highest average R^2^ values (0.932 and 0.924, respectively; Table 2) were also achieved with these spectral bands. Overall, these results are superior to those obtained when training from scratch. For example, compared to training from scratch with RGB bands (RMSE: 8.83 ± 0.29), fine-tuning represents a 16.8% improvement; even compared to the best scratch-training result (RMSE = 8.18 with GGG bands), this is an improvement of about 10%. Thus, the features learned by the pre-trained RESNET-18 proved useful for the RSQI prediction task and enhanced performance compared to training from scratch. Fine-tuning a pre-trained network also improved generalizability. As with training from scratch, good results were obtained regardless of the spectral bands used, but the RGB and RGN bands produced the best outcomes, while the NNN bands performed the worst. For all subsequent experiments in this study, the fine-tuned network with RGB spectral bands and a four-view input was used.

**Fig. 8:**
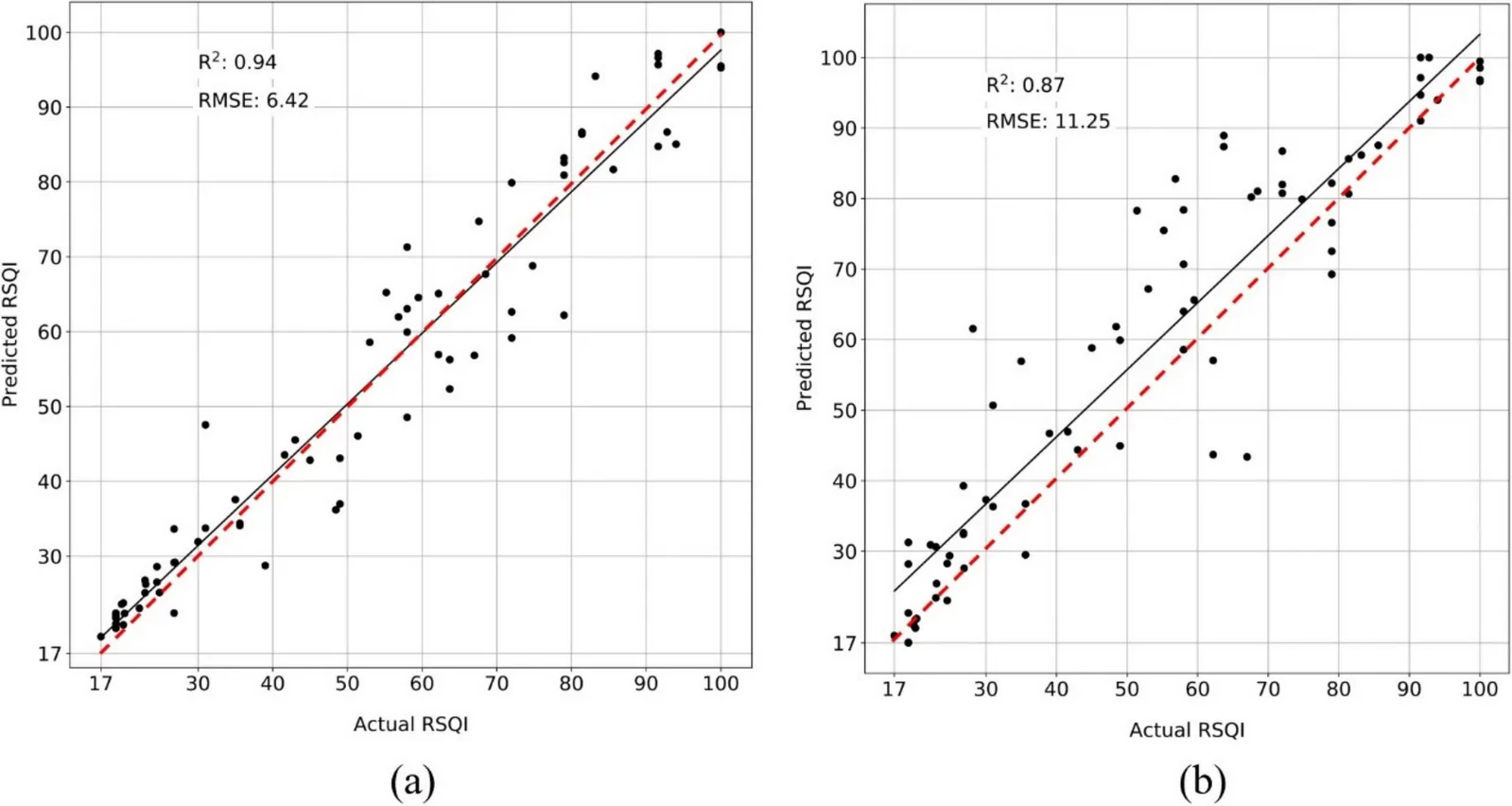
RSQI predictions from the best MVDCNN-based model (**a**) and from the object-based classification (OBC) method (**b**) as a function of field-measured index values. Each point represents the RSQI prediction for a riparian strip in the test dataset (*N* = 75). The red dashed line indicates the 1:1 slope (b = 1)

Figure 8a presents the predictions generated by the best model, that is, the optimal cross-validation iteration using RGB bands and four input views, for each riparian buffer in the test dataset. RSQI values below 30 were estimated with high accuracy (RMSE = 5.26). Predictions for values between 30 and 80 were more variable (RMSE = 8.09), while an RMSE of 6.11 was obtained for values between 80 and 100.

### Testing the model on simulated riparian strips

**Fig. 9:**
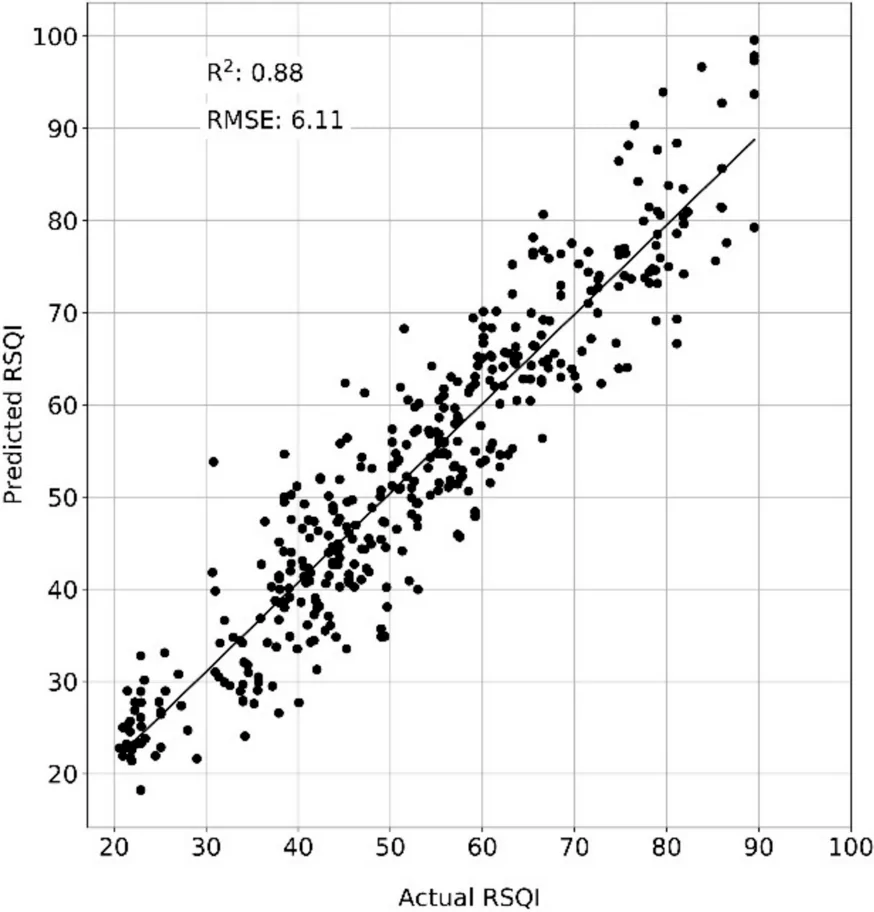
Model predictions when four views from two riparian strips are combined (N = 400). The reference RSQI value is the average of the two riparian strips

A series of cross-validation runs were performed to measure the model’s response to inter-image variability in land cover. For this simulation, four views each from two different riparian strips in the test set—a total of eight views—were used as network inputs. The ground-truth RSQI value was defined as the average of the two riparian strips. The view dataset was constructed by randomly selecting all possible combinations of RSQI classes, e.g., very poor–poor (RSQI 17–21, RSQI 21–40), poor–average (RSQI 17–21, RSQI 40–60), poor–good (RSQI 17–21, RSQI 60–80), and so forth. The results from 400 simulations are presented in Fig. 9.

As shown in Fig. 9, the model is able to account for the individual characteristics of a set of views when making predictions, even when the views are not from the same riparian strip. In this scenario, the R^2^ value is only 0.06 lower (0.87 obtained vs. 0.93 on the actual test dataset), while the RMSE is 0.35 higher (6.6 vs. 6.95 on the actual test dataset) compared to the original test set.

**Fig. 10:**
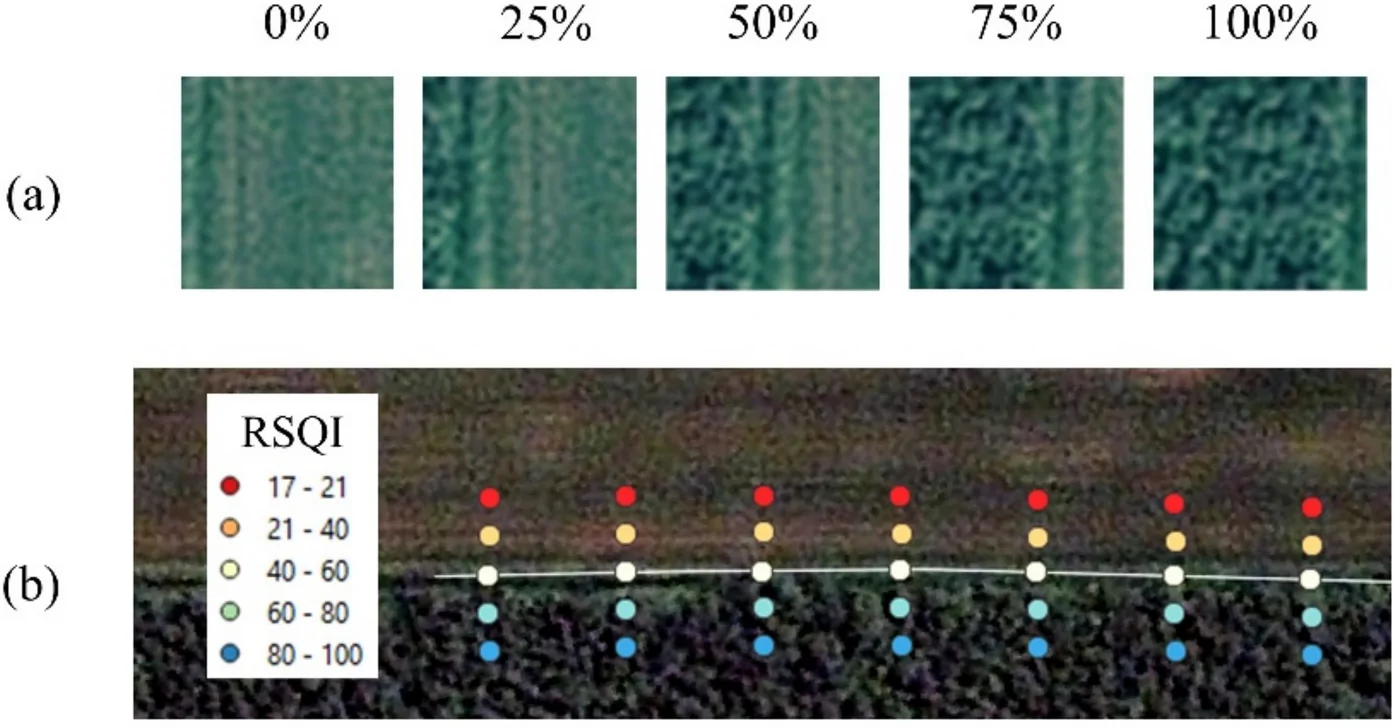
Example of a dataset used to evaluate an RSQI gradient. (**a**) Views composed of varying percentages of forest and agricultural land. (**b**) The riparian buffer at the forest–crop boundary shown in vector format (white line) each point indicates the center of an extracted view

**Fig. 11:**
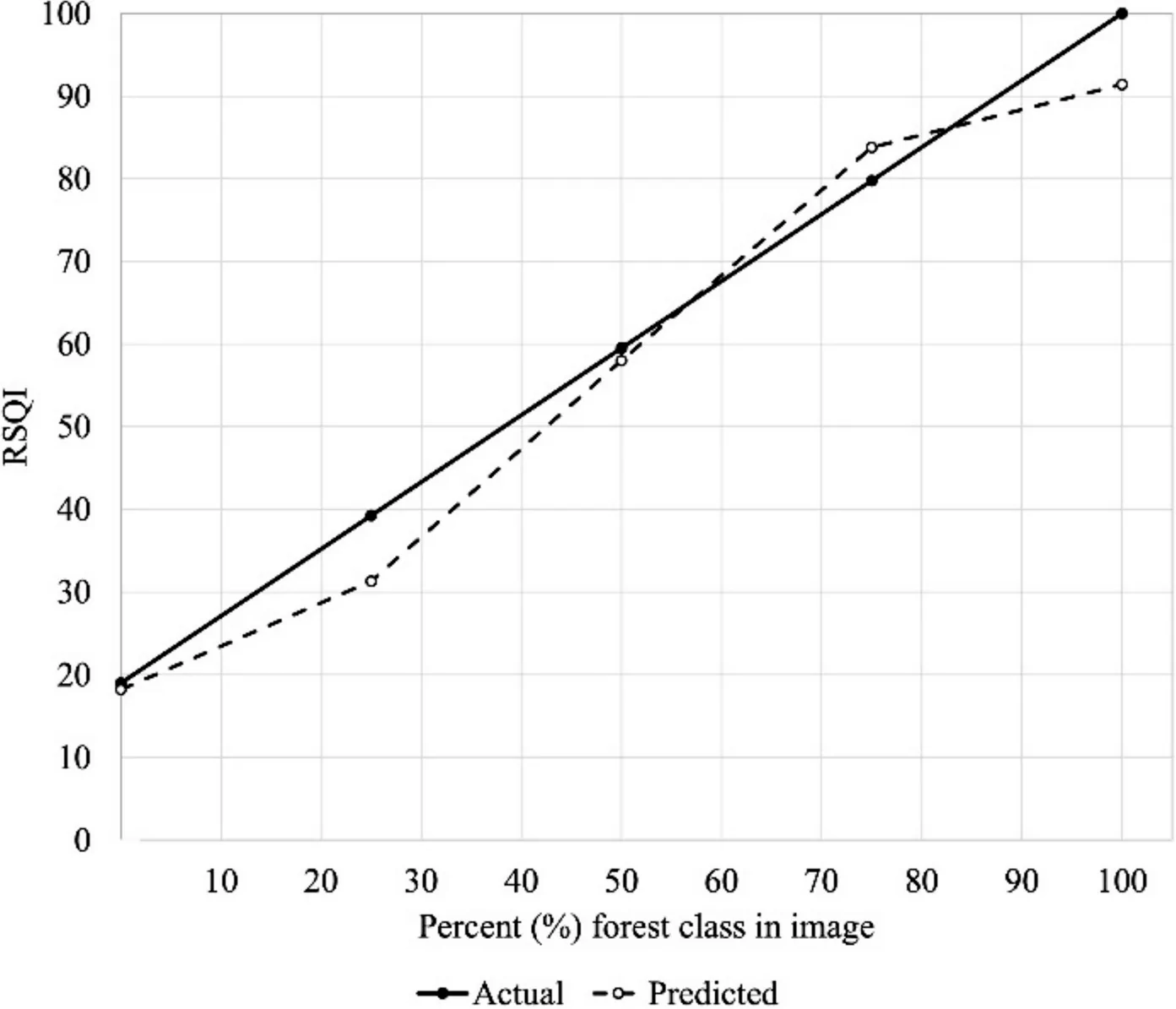
Five model predictions of RSQI as a function of the percentage of forest present in each view. The solid line represents the RSQI calculated from Eq. 1

The model’s response to intra-image variability in land cover was also assessed. For this purpose, a pseudo-riparian strip was delineated as a vector at the boundary between forest and agricultural land. Views were extracted along this vector, with each view (centered at the location shown as a dot in Fig. 10b) containing a defined proportion of forest and agricultural land-use classes. An estimated RSQI value was calculated for each view using Eq. (1). The analysis was repeated by shifting the riparian buffer location laterally, resulting in views containing 100%, 75%, 50%, 25%, and 0% forest area; the corresponding crop proportion was calculated as 100% minus the forest area. These two land-use classes represent a significant portion of the riparian strips in the dataset, which justified their selection for this test. An example of the resulting views is shown in Fig. 10a, and the prediction results are presented in Fig. 11.

Again, the trained model demonstrates sensitivity to intra-image variation in land cover represented by forest and agricultural land. The RSQI prediction errors (RMSE) range from 8.6 for views with 100% forest to 0.8 for views with 0% forest, values that are comparable to those observed on the test set.

### Object-based classification

For the object-based classification, overall accuracy across the eight land cover classes was 73% on 100 validation objects, with an overall kappa coefficient of 0.68 (Supplementary Table 1). Notably, the forest, herbaceous, pasture, and crop classes attained kappa coefficients of 0.79, 0.82, 0.74, and 0.57, respectively. These classes are the most commonly found within the first ten metres of riparian strip width and were considered in RSQI calculation. Excellent producer’s accuracies were also observed for the crop and forest classes (95% and 93.3%, respectively). In contrast, the shrub class was not correctly classified, but it is sparsely represented in the dataset. It is also worth noting that although the bare soil class yielded a kappa coefficient of 0.52, it is most often confused with infrastructure and crop classes, which have nearly identical weights in the RSQI (1.9, 1.7, and 1.9, respectively), thereby minimizing their influence on RSQI determinations.

**Fig. 12:**
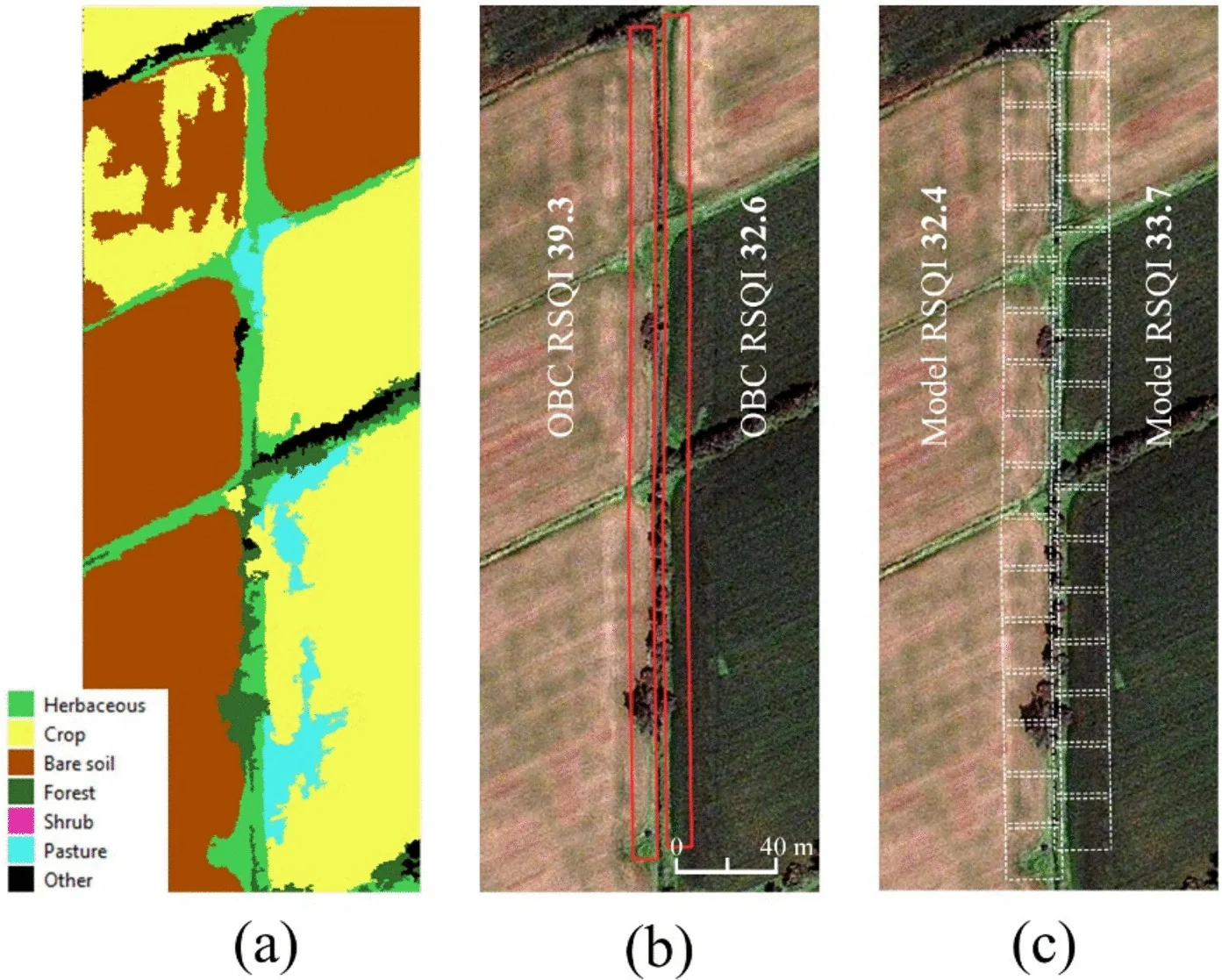
Representative object-based classification and RSQI predicted values. (**a**) Result of the OBC on the Pleiades image; (**b**) RSQI values calculated from the OBC with the 10 m buffer zone used for the calculation shown in red; and (**c**) RSQI predictions from the best MVDCNN-based model, with footprints of the input views indicated in white. The field-measured RSQI was 26.8 for both stream banks

An example of the classification results is shown in Fig. 12a, where some agricultural land was incorrectly assigned to the “pasture” class (cyan) for two riparian strips in the test dataset. In this specific case, the misclassification led to a small difference in the calculated RSQI (32.6 instead of 30.6, if correctly classified; see Fig. 12b). Native herbaceous plants, typically appearing as long and narrow objects in the satellite imagery, were generally well identified (light green). Discriminating these from crops is crucial given their distinctly different weighting factors (5.8 for herbaceous plants vs. 1.9 for crops). Overall, visual analysis of the results indicated good classification quality, particularly for the dominant land-cover classes in the dataset (i.e., forest, herbaceous, pasture, and crop).

The RSQI results obtained using object-based classification (OBC) are presented in Tables 1 and 2. For the 75 riparian strips in the test set, the OBC approach achieved an R^2^ of 0.87 and an RMSE of 11.25. Figure 12 provides a representative example from the test dataset, comparing the OBC method with our MVDCNN model. In this example, the average RSQI obtained with OBC for the two riparian strips is 35.95 (Fig. 12b), while the trained MVDCNN (Fig. 12c) predicted an average RSQI of 33.05, which is closer to the validated field data value of 26.8 for both strips.

## Discussion

A multi-input architecture was employed in this study because riparian strips, which can extend for many metres, cannot be adequately represented by a single 46 × 46 pixel image. To determine the optimal configuration, we evaluated networks with 1 to 16 simultaneous input views during training. Results obtained with a single input view (see Fig. 7), equivalent to a standard RESNET-18, demonstrate that this DCNN architecture is capable of learning relevant features. This outcome with a single view can be attributed to the homogeneous nature of riparian buffer strips in agricultural areas. However, the regression slope achieved with a single view was less accurate and failed to capture the full range of RSQI values as effectively as configurations with multiple input views. The MVDCNN architecture with four input views achieved better results, lowering RMSE by 0.25 (or 3.3%) on the test set and yielding a regression slope that more closely matched the linear variation of the RSQI index. This also suggests that it provides sufficient spatial coverage to capture the variability along a riparian strip segment while avoiding redundant information that would be introduced with more views. Nonetheless, for the sake of simplicity and ease of adaptation, one could use a widely adopted model with pretrained weights, such as a RESNET or EfficientNet (Tan et al., 2020), that operates on single images, expecting good if not optimal results. It would also be valuable to analyze the effectiveness of single-view training in more heterogeneous environments, such as partially populated areas, rocky ground surfaces, or regions experiencing extensive deforestation.

The training results using different spectral bands provided valuable insight into the features leveraged by the MVDCNN for this correlation task. Notably, training from scratch with a single spectral band—either blue (B) or green (G)—outperformed models using other spectral combinations, including all four available bands (see Tables 1 and 2). This suggests that texture, shape, and pattern information are crucial, as both poor- and good-quality riparian strips can be covered with healthy vegetation (trees, crops, grasses), resulting in similar spectral signatures. Differentiating these plant types is essential for riparian buffer characterization, making texture and shape key attributes. The use of very high spatial resolution imagery provides sufficient detail for the network to extract these features. The significance of texture may also explain the poorer results observed when using all four spectral bands, which contrasts with the findings of Mahdianpari et al. (2018), where models with more spectral bands achieved better accuracy for complex land-cover mapping. Consequently, using RGB-only imagery, which is typically less expensive and more widely available, emerges as a practical solution for integrating this approach into characterization efforts. Conversely, higher spectral resolution sensors with similar spatial resolutions could potentially offer improved characterization of vegetation features (Mariotto et al., 2013), though this may come at the expense of not fully exploiting the capabilities of well-known pre-trained networks. Nevertheless, the spectral and spatial information necessary to differentiate other land cover features, such as bare soil, buildings, and roads, is present in all four Pleiades sensor bands (Li et al., 2016).

The two training modes employed in this study (training from scratch versus using a pretrained RESNET-18) highlighted the importance of considering both the size and diversity of the training dataset. Although the image categories in ImageNet differ greatly from those used here, the low-level features learned by RESNET-18 remain relevant for remote sensing applications. Thus, architectures pretrained on large datasets can be effectively adapted to satellite imagery (Marmanis et al., 2016; Penatti et al., 2015). Indeed, features generated in the initial network layers, typically related to edge or colour detection, tend to be generic and transferable across different types of images and networks. In contrast, deeper layers encode more complex and dataset-specific features. Fine-tuning the top two layers of the pretrained network leverages these previously learned features while allowing adaptation to the current task, reducing the risk of overfitting and requiring fewer adjustable parameters.

While the dataset used in this study is considerable in terms of image count (46,209 extracted patches), its diversity does not match that of larger benchmark datasets, preventing models trained from scratch from achieving the same performance as those that are pretrained. Notably, only results obtained with the inclusion of the Near-infrared (N) band were superior to fine-tuned models (RMSE 8.4% lower), likely due to the absence of this spectral band in ImageNet. These findings suggest that pretraining on large Earth observation datasets such as BigEarthNet (Sumbul et al., 2021) or SEN12MS (Schmitt et al., 2019) could provide more relevant features for remote sensing applications, especially with regard to the N-band.

RSQI calculation was also performed using an object-based classifier in order to compare the proposed model with a more traditional approach. As shown in Tables 1 and 2, this approach resulted in lower accuracy than the proposed method. Comparing the average RMSE achieved by the MVDCNN on the test dataset with that of the OBC, we observed an average improvement of 3.16 (28.1%) with fine-tuning and 2.66 (23.6%) with training from scratch. Similar improvements, with average RMSE reductions of around 15.5%, were observed on the validation folds. Despite its lower performance, the OBC approach still achieved respectable results; however, its accuracy depends strongly on the quality of object segmentation and the final determination of land-cover classes. Additionally, this process requires photo-interpretation skills. Finally, the RSQI calculation method based on a strict buffer zone is less flexible than the MVDCNN-based approach, which can incorporate a broader spatial context.

A direct comparison with other results in the literature is not entirely fair, as correlations in those studies are often established between different indices (e.g., RSQI to QBR). In contrast, the proposed method involves training a model directly on RSQI data for RSQI prediction. Nevertheless, to give an indication of the approach's potential, Pace et al., (2022) reported a normalized RMSE (nRMSE) of 0.34 and Pearson's r = −0.75 for the correlation between remote sensing metrics and the QBR index, whereas our model achieved an nRMSE of 0.089 (7.35 RMSE normalized over the 83-unit RSQI range) and r = 0.965 (R^2^ = 0.932). Our results also significantly outperform those reported by Saha et al., (2020) and Rivas-Fandino et al., (2022), who obtained Pearson's r values of 0.462 and 0.538, respectively. Applying a similar approach to the QBR index, which is frequently used in Europe, would be an interesting avenue for future research.

Evaluation of errors on the test dataset revealed several limitations of the trained models. First, riparian strips with RSQI values between 30 and 80 were more challenging for the model to predict accurately (see Fig. 8a). This may be due to the smaller number of training examples within this range (see Fig. 3a for dataset distribution), despite class balancing during training, and the fact that these strips exhibit the most heterogeneous land cover, making them more difficult to assess for both in situ evaluators and the model itself. Moreover, different land cover configurations can produce similar RSQI values, and not all such configurations may be equally represented in the training data. Second, the model sometimes tends to overestimate RSQI in the presence of trees. This limitation arises from the top-down perspective of satellite imagery: large tree canopies can obscure riparian strips that are predominantly covered by grass or crops, rendering these ground-level features unobservable in the images. To address, this issue, LiDAR and Radar data—capable of penetrating forest canopies—could be employed to reduce uncertainty in RSQI estimates in such vegetated environments.

A major impediment to model development is the subjective nature of the in situ RSQI measurements upon which DCNN training is based (Novoa et al., 2018). The accuracy of land-use estimates for each riparian buffer component ultimately depends on the individual assessor’s perspective at the time of the survey. In this study, field data were collected over three years by several teams across a large area. This inherent subjectivity, compounded by the use of different evaluators, introduces uncertainty into the dataset. For example, distinguishing between pasture/agricultural grassland (RSQI weight of 3.0) and natural grassland (weight of 5.8) can be problematic: for similar areas, these land covers were sometimes categorized differently depending on the evaluator. This confusion is understandable, as natural grasses are often present within agricultural grasslands, complicating interpretation. As a result, contextual information becomes an important part of the evaluation. To enable more replicable assessment and address this limitation, new characterization indices based solely on remote sensing products are being developed (Novoa et al., 2018). Another source of uncertainty in the training data arises from temporal discrepancies between satellite image acquisition and field campaigns (which may vary by 1–2 years), as well as differences in the timing within the season when each was conducted. Collectively, these sources of uncertainty introduce noise into the training dataset. Nevertheless, despite this limitation, the trained models successfully learned relevant features for RSQI determination, as evidenced by their superior performance on the curated test dataset compared to the validation dataset.

The empirical nature of the proposed approach also entails several inherent limitations, including training the model exclusively with satellite images of a specific spatial resolution (50 cm) and from a particular environment, namely, agricultural areas in southern Quebec. The training data is further constrained by the land cover configurations available in the L'Acadie River watershed, which is characterized by relatively limited land cover heterogeneity due to its agricultural dominance. Consequently, the dataset does not encompass all possible land cover configurations that could yield the same RSQI value. Moreover, all images used for model training were acquired in July, necessitating further testing to evaluate the model’s robustness to seasonal variability. Training with a temporal series of satellite images, rather than single-date images, could serve as an effective strategy to address this limitation. Application of the model to regions with substantially different ecological contexts, such as different crop types, stream characteristics, or riparian vegetation structure, would require retraining or fine-tuning with a representative dataset from the target region.

A few essential prerequisites must be met to implement the approach. First, an adequately sized characterization dataset is required, representative of the geography and environment where riparian buffer quality is to be assessed. This assumes that the parameters used for quality index calculation are largely derivable from remote sensing images. Second, vector data delineating the relevant watercourses are needed, typically available from government agencies. Finally, very high-resolution satellite imagery for the area of interest is required: one or more images from the same time period as the characterization data (for training) and additional images from the period(s) to be evaluated. As the approach is applied over time, the model can be incrementally improved with newly acquired images and characterization data, making it increasingly accessible and cost-effective.

While the MVDCNN approach bypasses the segmentation and land cover classification steps required by traditional RSQI mapping workflows, practical application does require specific technical expertise. The main advantage over traditional methods is eliminating dependency on object-based classification, which requires iterative parameter tuning through trial and error, and the need for expert photo-interpretation. However, model application requires programming skills and, ideally, machine learning expertise, particularly for fine-tuning the model to new contexts. This study presents the methodological approach; further software development would be needed to translate it into accessible tools for operational use by practitioners.

The developed model of this study was trained to predict the Riparian Strip Quality Index (RSQI) directly from satellite images. Tests on simulated riparian strips (Figs. 9 and 11) demonstrate that the model captures variations in land cover and is particularly sensitive to the presence of trees. From a practical perspective, this model could facilitate annual monitoring of riparian buffer status, thereby enhancing decision-makers’ ability to identify areas in need of intervention. It also enables rapid assessment of riparian buffer quality in regions not yet covered by field campaigns, providing valuable decision support for determining where such campaigns are warranted. The MVDCNN architecture is flexible regarding the number of input views, so its application is not limited by river length. Overall, the potential of this approach demonstrated in this study suggests it could serve as a viable alternative for the characterization of riparian buffer strips.

## Conclusions

This research proposed a straightforward methodology for characterizing riparian buffer strips in agricultural landscapes, combining deep convolutional neural networks (DCNNs) with high-resolution satellite imagery and leveraging an extensive RSQI field dataset. To our knowledge, this is the first direct application of DCNNs to riparian buffer assessment, marking a significant contribution to the field. The adapted MVDCNN architecture, incorporating RESNET-18, achieved strong correlations between satellite imagery and RSQI metrics. Notably, the selection of spectral bands for network training had minimal impact, with all combinations delivering comparable results. In addition to being more accessible in terms of required remote sensing or photo-interpretation skills, this method outperformed traditional object-based classification approaches in correlating imagery with field RSQI data.

As riparian strips are often narrow and irregularly shaped, a single view cannot capture them entirely without including extraneous features. The choice of the MVDCNN architecture was therefore motivated by the need to utilize multiple input views corresponding to a single index value. Incorporating four input views led to a modest improvement in average RMSE on the test set compared to using only one patch. In relatively homogeneous settings, such as those in this study, a traditional model based on a single input view may suffice. Nonetheless, the flexible design of the MVDCNN enables inferences with varying numbers of views, making it adaptable to different riparian strip configurations.

Looking ahead, increasing the public availability of field characterization datasets would facilitate the development of data-intensive approaches by a broader range of research teams. This could spur the creation of models that are more efficient, versatile, and resilient across diverse and complex environments. Ultimately, such advancements would pave the way for large-scale, highly recurrent monitoring of riparian buffer conditions with minimal need for on-site validation, supporting more effective ecosystem management practices.

## Supplementary Information

Below is the link to the electronic supplementary material.

Supplementary file1 (DOCX 33 KB)

## Data Availability

Partial data presented in this study are available on request from the corresponding author.

## Declarations

All authors have read, understood, and have complied as applicable with the statement on “Ethical responsibilities of Authors” as found in the Instructions for Authors.
