# Supplementary material for: A CNN-based approach to riparian buffer strip quality monitoring in agricultural areas using satellite imagery
Source: Environ Monit Assess. 2026 Sep 5;198(10):1042. doi: 10.1007/s10661-026-15760-w (PMC13546348; doi:10.1007/s10661-026-15760-w)
Supplement: Supplementary file 1 [file 10661_2026_15760_MOESM1_ESM.docx]

**A CNN-based Approach To Riparian Buffer Strip Quality Monitoring in Agriculture Areas Using Satellite Imagery – *Environmental Monitoring and Assessment***

Samuel de la Sablonnière ^a^, Samuel Foucher ^b^, Yacine Bouroubi ^b^, Philippe Vigneault ^a^ and Etienne Lord^a^

*^a^ Saint-Jean-sur-Richelieu Research and Development Centre, Agriculture and Agri-Food Canada, Saint-Jean-sur-Richelieu, Canada, ^b^ Department of Applied Geomatics, University of Sherbrooke, Sherbrooke, Canada*

**Corresponding author contact details :**

Samuel de la Sablonnière

Remote sensing and Geomatics Specialist, Technology Specialty Support Group in Geomatics and Precision agriculture , Saint-Jean-sur-Richelieu Research and Development Centre, Agriculture and Agri-Food Canada, 430 Gouin Boulevard, Saint-Jean-sur-Richelieu, QC J3B 3E6, Canada

E-mail: [samuel.delasablonniere2@agr.gc.ca](mailto:samuel.delasablonniere2@agr.gc.ca)

**Supplementary**

**Supplementary Table 1. Confusion matrix from the object-based land-cover classification (OBC)**

|  | | Bare soil | Herbaceous | Crop | Pasture | | Forest | | Infra-structure | Shrub | Other | ^1^User’s acc. (%) | *k* |
| --- | --- | --- | --- | --- | --- | --- | --- | --- | --- | --- | --- | --- | --- |
| Bare soil | | **8** | 1 | 0 | 0 | 0 | | 3 | | 0 | 2 | 57.14 | 0.519 |
| Herbaceous | | 0 | **11** | 0 | 0 | 1 | | 1 | | 0 | 0 | 84.62 | 0.821 |
| Crop | | 1 | 0 | **19** | 6 | 0 | | 0 | | 3 | 0 | 65.52 | 0.569 |
| Pasture | | 0 | 1 | 1 | **11** | 0 | | 0 | | 1 | 0 | 78.57 | 0.742 |
| Forest | | 0 | 1 | 0 | 0 | **14** | | 0 | | 2 | 0 | 82.35 | 0.792 |
| Infrastructure | | 2 | 0 | 0 | 0 | 0 | | **7** | | 0 | 0 | 77.78 | 0.750 |
| Shrub | | 0 | 0 | 0 | 0 | 0 | | 0 | | **0** | 1 | 0.00 | -0.064 |
| Other | | 0 | 0 | 0 | 0 | 0 | | 0 | | 0 | **3** | 100.0 | 1.00 |
| ^1^Producer’s acc. | 72.7% | | 78.6% | 95.0% | 64.7% | 93.3% | | 63.6% | | 0.0 % | 50.0% |  |  |
| Overall accuracy | 73.0 % | | | | | | | | | | | | |
| Overall *k* | 0.68 | | | | | | | | | | | | |

^1^Producer's accuracy: number of correctly classified reference sites / total number of reference sites. images; User's accuracy: number of correctly classified sites / total number of classified sites.
